# Measuring the effect of intimate partner violence on health-related quality of life: a qualitative focus group study

**DOI:** 10.1186/1477-7525-5-67

**Published:** 2007-12-19

**Authors:** Eve Wittenberg, Manisha Joshi, Kristie A Thomas, Laura A McCloskey

**Affiliations:** 1 Heller School for Social Policy and Management, Brandeis University, Waltham, MA, USA; 2 School of Social Policy and Practice, University of Pennsylvania, Philadelphia, PA, USA; 3 Merrill-Palmer Skillman Institute, Wayne State University, Detroit, MI, USA

## Abstract

**Background:**

Health related quality of life (HRQOL) can be measured by a wide range of instruments, many of which have been designed for specific conditions or uses. "Preference-based" measures assess the value individuals place on health, and are included in economic evaluations of treatments and interventions (such as cost effectiveness analysis). As economic evaluation becomes more common, it is important to assess the applicability of preference-based health related quality of life (HRQOL) measures to public health issues. This study investigated the usefulness of such instruments in the context of intimate partner violence (IPV), a public health concern that that can seriously affect quality of life.

**Methods:**

The study consisted of focus groups with abused women to determine the aspects of life affected by IPV, and an analysis of existing HRQOL measures. Eight focus groups (n = 40) were conducted in which participants discussed the domains of health affected by IPV. Results were content analyzed and compared with the domains of health included in four commonly-used, preference-based HRQOL measures.

**Results:**

The average focus group participant was 43 years old, unemployed, African American, with 3 children. Domains of health reported to be affected by IPV included physical functioning, emotional and psychological functioning, social functioning and children's functioning. Psychological health was the most severely affected domain. The Short Form 36, the Health Utilities Index, the EuroQol 5D, and the Quality of Well-being Scale were found to vary in the degree to which they include domains of health important in IPV. Psychological health is included to a limited extent, and the spill-over effect of a condition on other family members, including children, is not included at all.

**Conclusion:**

Emotional and psychological health plays an important role in the overall HRQOL of abused women but is relatively underemphasized in preference-based HRQOL measures. This may lead to an underestimation of the impact of partner violence on HRQOL when using these measures and in economic evaluations that rely thereon. Holistic measurement approaches or expanded measures that capture the far-reaching effects of IPV on HRQOL may be needed to accurately measure the effect of this condition on women's health.

## Background

Intimate partner violence (IPV) has wide-ranging and oftentimes unmeasured effects on health and quality of life[1]. IPV is relatively common compared with other conditions that affect the health of women: one in four women in the United States reports experiencing violence from an intimate partner over her lifetime, and each year at least 1.5 million women are assaulted by intimate partners[2]. The documented health effects of partner abuse range from severe injury or even death to somatic complaints[3]. Compared with women who have not been abused, abused women report more health symptoms such as headaches and gynecologic discomfort[4,5], and they are more likely to be diagnosed with specific conditions such as irritable bowel syndrome, arthritis, and a range of serious conditions entailing hospitalization [4-6], and are more likely to be depressed[7].

The effect of IPV on quality of life has been less studied. Measures of quality of life are useful for understanding the subjective effect of health on individuals, including the perception of well-being that accompanies specific symptoms or diagnoses. Quality of life is also useful in outcome evaluations, providing a quantitative measure of the effect of a condition on individuals' lives and thereby a measure of the benefit of preventing or intervening in that condition. In particular, health related quality of life (HRQOL) is often used in economic evaluations as a component of the benefit derived from an intervention, which demonstrates effectiveness as well as cost-effectiveness when compared with costs.

Health-related quality of life is a general term that describes the overall impact of a disease, illness or condition on the health and well-being of the affected individual. HRQOL can *describe *an individual's health and well-being in terms of symptoms and functioning, or it can reflect how an individual *values *a particular state of health, meaning how much they like or dislike being in that particular state of health and well-being. This value-focused measure of HRQOL is termed "preference-based" because it measures an individual's *preference *for a health state, as opposed to an individual's *description *of the state[8]. Preference-based measures of HRQOL can be used in aggregate to reflect the value that society as a whole associates with being in a particular state of health[9]. Such values are often used in decision making about prioritization of resources across competing programs and interventions, to answer questions such as should we spend resources to prevent IPV versus automobile accidents, to treat HIV versus cancer? While the association between intimate partner violence and specific health complaints has been identified, we know little about the effect of IPV on overall health related quality of life and the value that individuals and society place on the effects of IPV on women's lives[1].

There exist a wide variety of methods to measure preference-based HRQOL. Approaches vary from directly questioning individuals with experience with a particular condition about how they value it, to two-part methods in which (1) an individual who has experienced a particular condition describes it, and then (2) a separate set of values is applied to these descriptions[8,9]. This two-part method makes use of standardized instruments to collect the descriptive information about a health condition from people who have experience with it, prior to assigning values to these descriptions. Commonly-used instruments include the Short-Form 36 (SF-36) and its variations (e.g., SF-12, SF-6D [10-12]), the Health Utilities Index (HUI [13], the EuroQol 5D (EQ-5D [14]), and the Quality of Well-Being Scale (QWB [15]). These instruments elicit descriptive information about the effect of a disease or condition on various domains of health, ranging from things like vision and dexterity to social functioning and vitality. These domains are intended to capture the range of aspects of health that can be affected by disease, and that are important to quality of life. (Note: While the HUI, EQ-5D and QWB were designed as preference-based measures, meaning they were designed to capture the value that people place on being in a particular state of health or having a particular condition, the SF-36 was originally designed to measure health status, and methods have subsequently been developed to translate it into a preference-based measure [16-19])

While focusing individuals' attention on specific domains of health may be helpful to elicit the full range of impact of a condition, it may also exclude effects in domains not specifically queried. We hypothesized that such a situation may exist in the case of intimate partner violence: that certain domains of health affected by IPV may not be included in these commonly-used HRQOL measures, and thus the effect of IPV on HRQOL may be misestimated when using such instruments. The purpose of this research was therefore to expand our understanding of the domains of health affected by IPV to accomplish two goals: (1) to inform the health ramifications of partner violence, and (2) to inform the measurement of the effect of IPV on preference-based health related quality of life. The ultimate goal of this research was to improve the measurement of HRQOL by identifying potential sources of measurement bias that may result from the inclusion and exclusion of domains in instruments. We report here on the results of focus groups of abused women discussing the health and quality of life effects of IPV, and a review of preference-based health related quality of life instruments designed to measure these effects.

## Methods

In this qualitative study we conducted focus groups with abused women to measure perceptions of the effect of intimate partner violence on health and quality of life. We compared results from these groups with existing preference-based HRQOL instruments to assess the adequacy of these instruments to capture the aspects of health and quality of life affected by IPV. The study was approved by the University of Pennsylvania Institutional Review Board.

### Sample

Women were recruited via flyers posted in domestic violence shelters and service providers and their surrounding areas in greater Philadelphia, PA. Potential participants were screened by phone prior to participation in the groups. Inclusion criteria included 18 years of age or older, English-speaking, having been in a relationship with a man and having experienced "physical abuse or severe control from a male partner" in the past 12 months. Fifty-nine women were recruited to the study of which 40 actually participated (some did not appear at their scheduled group and some exceeded the intended sample size). Although not part of the recruitment plan, snowball sampling occurred among women who attended a group and their friends and relatives. All women in the study provided written, informed consent for participation.

### Focus group procedures

Eight focus groups were conducted in Philadelphia in March and April, 2006. Each group lasted between 60 and 90 minutes, was led by a trained moderator using a semi-structured discussion protocol, and was audio taped. Participants' transportation costs were reimbursed and they were each remunerated $50 for their time. Childcare was provided on site and a licensed social worker was available to women during and after the groups.

### Data collected

Demographic and abuse data were collected individually from each woman including age, race, employment and marital status, and number of children, and the Women's Experience with Battering Scale (WEB[20]) and a modified version of the Conflict Tactics Scale (CTS [21]). The WEB measures psychological terror or battering in a current relationship and ranges in score from 10 to 60 where 20 or higher is indicative of battering. The CTS measures frequency and severity of physical, emotional and sexual abuse in both current and former relationships and a positive score indicates current abuse. While focus group discussions were wide-ranging and followed topics mentioned during the conversations, moderators focused the discussions on women's experience of IPV and the impact on their physical and emotional health and well-being. Descriptions of health related quality of life instruments were obtained from the literature and published sources.

### Analysis

Demographic and abuse data were summarized with descriptive statistics. The focus group audiotapes were transcribed verbatim by three research assistants, none of whom had contact with the participants. Transcription reliability was checked in a random sample of sections by the authors (KT and MJ) revealing discrepancy rates of 9.7% and 2%. Most discrepancies related to wording differences or word omissions and very few changed the actual meaning of the women's conversations. Transcripts were coded by one author (EW) and a research assistant for the domains of health and quality of life mentioned. Each mentioned area in which health or quality of life was affected was recorded, and affected areas were categorized into domains of life and health. We mapped the affected areas onto the domains used in four existing health related quality of life instruments and added other domains for those areas that were unrepresented in existing measures.

## Results

### Sample characteristics

The average participant was 43 years old, African American, single, unemployed and had three children (Table 1). The majority of women reported current abuse by either the Conflict Tactics Scale (mean score = 111) or the WEB (mean score = 32). Four women had a CTS score of zero and four had scores over 300 (three of the four with zero CTS scores had scores that indicated battering on the WEB, and the fourth had a score very close to the battering threshold). Seventy-five percent of the women met the WEB criterion for psychological battering. Over 40% of the women reported IPV in prior years.

**Table 1:** Characteristics of sample: 40 women in 8 focus groups

Age: mean years (sd)	42.6 (10.6)
Range	18–64
Race/Ethnicity n (%)	
African American	30 (79%)
White	3 (8%)
Latina	2 (5%)
Multiracial/ethnic	3 (8%)
Employment n (%)	
Full-time	5 (13%)
Part-time	6 (15%)
Unemployed	28 (72%)
Marital Status n (%)	
Married	16 (41%)
Divorced	4 (10%)
Single	19 (49%)
Has at least 1 child n (%)	31 (78%)
Number children: mean (sd)	3.3 (1.7)
CTS Score: mean (sd)	111 (122)
Range	0–380
WEB score: mean (sd)	32 (15)
range (%> = 20)	0–50 (75%)
IPV prior to last year n (%)	17 (43%)

%s may not sum to 100 due to rounding.

Employment and marital status missing for 1 woman each; Race missing for 2.

CTS = Conflict Tactics Scale

WEB = Women's Experience with Battering Scale

IPV = Intimate partner violence

### Focus group discussions

Women reported very severe physical, emotional and psychological abuse, ranging from beatings, chokings, and burns, to stalking, poisoning, imprisonment, rape, and abuse of their children, pets and property (for more detail on reports, see [22]). They reported that these experiences affected their physical and emotional health in four general categories: physical functioning, emotional and psychological functioning, social functioning, and their children's functioning (Table 2). Almost all of the women reported that the emotional and psychological dimensions of health were most significantly affected by abuse, and they mentioned more emotional and psychological than physical sequelae of abuse. The abuse manifested in physical terms beyond the injuries inflicted upon them, in symptoms such as headaches, insomnia, fatigue and high blood pressure (Table 3). While some of these conditions may have an etiology independent of the abuse, the women reported a self-perceived association between the violence and their physical symptoms.

**Table 2:** Reported domains of life affected by intimate partner violence

Physical Functioning
E.g., headaches, insomnia, vomiting, fatigue/lethargy, heart palpitations, high blood pressure, addiction relapse
Emotional and psychological functioning
E.g., crying, sadness, anger, aggression, loneliness, worry and anxiety, depression, fear, helplessness and powerlessness, resignation, confusion, shame, embarrassment, stress, paranoia, flashbacks and nightmares.
Social functioning
E.g., isolation from friends, family, religious groups; ostracization by family, friends and church; lack of confidence in police and service providers; inability to work (due to interference by abuser or from poor health).
Children's functioning
E.g., aggression, anger, fighting; self-destructive behavior; nail biting; stuttering; gambling; substance abuse; poor school performance.

**Table 3:** Reported Physical and Emotional/Psychological Symptoms of Abuse

**Physical symptoms**
Addiction relapse
Asthma
Fatigue
Graying hair
Headaches (migraines)
Heart attack
Heart palpitations
High blood pressure
Insomnia
Lethargy
Voice change
**Emotional/psychological symptoms**
Anger
Anxiety, nervousness
Apathy
Becoming abusive herself
Confusion
Crying
Depression
Difficulty focusing
Fear, including:
Fear of particular places (e.g., where abuse occurred)
Fear of losing children, fear of abandoning children
Fear of repercussions
Fear for life
Fear that medications will be tampered with
Fear of becoming abusive herself
Fear of impact of stress on other co-morbid conditions
Feelings of failure
Feelings of worthlessness
Feeling "on edge"
Feeling rejected/abandoned
Feeling trapped, stuck
Flashbacks
Frustration
Guilt
Helplessness/powerlessness
Hyper-awareness
Loneliness
Loss of self-esteem/loss of confidence
Loss of trust in others
Mood swings
Nightmares
Over-eating
Panic
Paranoia
Resignation
Sadness
Self-blame
Self-consciousness
Self-hatred
Shame, embarrassment
Stress
Suicide attempts
Suicidal ideation
Worry

The women's reported emotional and psychological symptoms include many of those of post-traumatic stress disorder, such as hyper vigilance, flashbacks and nightmares, fear, anger and aggression (Table 3). In every group, women discussed experiences of shame and embarrassment resulting from their abuse, and feelings of loneliness, isolation, helplessness and depression were common. They emphasized the importance of their loss of freedom and control over their lives, exemplified by experiences of interference at work (e.g., the abuser disparaging the woman to her employer or injuries preventing her from working) and being physically prevented from contacting friends or relatives. Most women reported that the effect on their quality of life of the emotional and psychological symptoms that resulted from IPV was more important than that from the physical symptoms.

In addition to the direct effect of abuse on the women's health and quality of life, women reported that their own quality of life suffered from their knowledge of the negative effect that witnessing abuse had on their children. Women reported significant changes in their children's behavior which in turn resulted in an exacerbation of the women's physical and emotional symptoms, including increased worry, guilt, anxiety and depression. While women reported that some children also experienced direct abuse from the intimate partner, many reported on the effect of witnessing their mother's abuse.

### HRQOL instruments

The SF-36, HUI, EQ-5D and QWB instruments are limited in their inclusion of emotional or psychological aspects of health (Table 4). The longer instruments and those that measure more domains of health tend to include more psychological and emotional attributes of health, such as the SF-36, which with eight domains measures "social functioning," "role-emotional," and mental health. The Health Utilities Index (Mark 3) includes "emotion" as one of eight domains and the EuroQol includes "anxiety/depression" as one of five domains. The Quality of Well Being Scale does not directly measure emotional or psychological health but includes "social activity" which may encompass some aspects of emotional and psychological health. Both the SF-36 and the EuroQol include holistic assessments of health that might further elicit emotional and psychological components of health, the SF-36 through "vitality" and "general health" domains and the EuroQol through a visual analog scale (a 0–100 scale that elicits a numerical representation of self-perceived overall health). The visual analog scale in particular allows for an encapsulation of all affected domains, but it does not identify or differentiate among domains, thereby adding sensitivity to the overall assessment but lacking specificity.

**Table 4:** Domains included in Health-related Quality of Life instruments

**Short Form 36 [11, 12]**
Physical functioning
Role–Physical
Bodily Pain
General Health
Vitality
Social functioning
Role–Emotional
Mental health
**Health Utilities Index (Mark 3) [13]**
Vision
Hearing
Speech
Ambulation
Dexterity
Emotion
Cognition
Pain
**EuroQol 5D [14]**
Mobility
Self-care
Usual activities
Pain/discomfort
Anxiety/depression
*Visual analog scale*
**Quality of Well Being Scale [15]**
Mobility
Physical activity
Social activity
Symptoms list

## Discussion

For women included in this study, the effects of violence perpetrated by an intimate partner were concentrated in the emotional and psychological domains of health despite the apparent physical effects of abuse. Fear, control and power played significant roles in women's negative outcomes from IPV. Furthermore, children's experience of witnessing IPV had a significant impact on their mothers' HRQOL, beyond the effect on the children themselves. Existing preference-based measures of HRQOL focus more on the physical domains of health than the emotional and psychological domains, contrary to those which were most severely affected among the women in our study. The importance of children's quality of life to these mothers' HRQOL suggests the need for a wider conception or definition of HRQOL, possibly focusing on the family unit as a whole[23,24]. Measures of HRQOL developed for physical illnesses may underestimate the effect of IPV for some women, and resource decisions made on this basis may possibly be misguided. To adequately capture the HRQOL impact of IPV for these women, HRQOL measures may need to include greater and broader focus on psychological outcomes, or possibly focus on holistic measures that include all aspects of self-perceived quality of life.

Generic HRQOL instruments are extremely valuable because of their ease of use and adaptation to many varying conditions. Preference-based measures of HRQOL are particularly important because of their role in economic evaluations upon which resource allocation decisions are often based. The benefit attributed to particular interventions is oftentimes measured with preference-based measures of HRQOL, meaning that accurate assessment of benefits is dependent upon accurate measurement instruments. In this context, the decision to allocate resources to IPV prevention or intervention efforts may hinge upon accurate measurement of the impact of IPV on quality of life and hence the benefit that would accrue from preventing or intervening in violence. While many of the existing preference-based HRQOL instruments were originally designed to measure the effect of medical conditions (e.g., [25]), they have been adopted for more general use because of their ease of use and comparability across conditions. The prevalent use of these measures requires that they be considered for a broader range of uses than may have been originally intended, including non-medical conditions such as IPV, and that they be adapted accordingly.

Researchers measuring HRQOL for any purpose should take care to choose measures that encapsulate the entirely of impact of a condition on health, and define health in an appropriate way for the condition under consideration[26]. The domains specified by each instrument define the aspects of life that are included and excluded in that assessment of HRQOL. When choosing among instruments, researchers should consider the aspects of health and life that are expected be affected by a condition. Notice should also be taken of the more general or holistic elements included in some instruments, such as the visual analog scale in the EuroQol and the general health measure in the SF-36, which may capture aspects of health not otherwise included in specified domains. These general measures may also be considered as a validity check of other domains included in a composite measure, or to inform the more subjective aspects of HRQOL. Our results suggest that for at least some women, measuring the effect of IPV on HRQOL may require a broader definition of health than is included in commonly-used, preference-based measures of HRQOL in order to adequately capture the entirely of their experience. Other health conditions may have similar impacts on aspects of life and health that are not included in generic HRQOL measures, and should be explored to obtain unbiased estimates of the burden of the condition/disease on those affected.

Of particular note are the effects of health conditions on individuals surrounding the index person or patient, including family members and caretakers[23,27]. Such effects are often difficult to measure, yet may make a significant contribution to the overall impact of a disease or condition. The "spillover" effect of children's health on their parents' quality of life is occasionally considered[28], as well as the effect of illness on siblings[23,29]. Measurement of the indirect connection between parent and child HRQOL that we observed in our sample, in which the health of the woman affects the child which in turn further affects the woman, is unprecedented in HRQOL measures. Our observation of the significant impact of children's distress on their mother's HRQOL suggests the need for the inclusion of a new domain in the measurement of HRQOL in this context, and potentially in others as well.

It is important to acknowledge that the data on which this research is based has extraordinary richness but accordingly limited generalizability. We spoke with 40 women, mostly African American, from one urban area. It is not known whether the emotional and psychological impact of violence differs by race or geographic location, so our results must be considered in context and with caution. And though our data are self-reported and unconfirmed by objective measures, we believe that self-report bias would tend toward underreporting of abuse and the impact thereof, so our results might be considered a lower bound of the effect. The qualitative reports of HRQOL by women in our focus groups are consistent with their CTS and WEB scores, providing some internal consistency in our data. Nevertheless, further research on abused women and the range of effects of violence on their lives would be a welcome addition to the sparse literature on outcomes of IPV.

## Conclusion

In conclusion, IPV has substantial effects on women's health related quality of life in areas that may not have been previously identified. Misunderstanding or underestimation of the impact of IPV on HRQOL could lead to inefficient allocation of resources from a health and social policy perspective of endeavoring to provide the greatest benefit from resources spent on prevention and intervention. Efforts to accurately identify and measure the impacts of IPV on women's health and quality of life may lead to more effective interventions and policy decisions.

## Abbreviations

(in order of appearance in manuscript)

IPV: Intimate partner violence;

HRQOL: Health related quality of life;

SF-36: Short-Form 36;

SF-12: Short-Form 12;

SF-6D: Short-Form 6 domains;

HUI: Health Utilities Index;

EQ-5D: EuroQol 5 domains;

QWB: Quality of Well-Being Scale;

WEB: Women's Experience with Battering scale;

CTS: Conflict Tactics Scale.

## Competing interests

The author(s) declare that they have no competing interests.

## Authors' contributions

EW and LAM conceived of and designed the study. MJ and KAT recruited subjects for and organized the focus groups. EW analyzed and interpreted the data. All authors participated in the conduct of the focus groups and all read and approved the final manuscript.
